# Inferior vena cava displacement during respirophasic ultrasound imaging

**DOI:** 10.1186/2036-7902-4-18

**Published:** 2012-08-06

**Authors:** David J Blehar, Dana Resop, Benjamin Chin, Matthew Dayno, Romolo Gaspari

**Affiliations:** 1 Department of Emergency Medicine, University of Massachusetts Medical School, 55 Lake Ave North, Worcester, MA, 01655, USA

**Keywords:** Inferior vena cava, Ultrasound, Technical errors

## Abstract

**Background:**

Ultrasound measurement of dynamic changes in inferior vena cava (IVC) diameter can be used to assess intravascular volume status in critically ill patients, but published studies vary in accuracy as well as recommended diagnostic cutoffs. Part of this variability may be related to movements of the vessel relative to the transducer during the respiratory cycle which results in unintended comparison of different points of the IVC at end expiration and inspiration, possibly introducing error related to variations in normal anatomy. The objective of this study was to quantify both craniocaudal and mediolateral movements of the IVC as well as the vessel's axis of collapse during respirophasic ultrasound imaging.

**Methods:**

Patients were enrolled from a single urban academic emergency department with ultrasound examinations performed by sonographers experienced in IVC ultrasound. The IVC was imaged from the level of the diaphragm along its entire course to its bifurcation with diameter measurements and respiratory collapse measured at a single point inferior to the confluence of the hepatic veins. While imaging the vessel in its long axis, movement in a craniocaudal direction during respiration was measured by tracking the movement of a fixed point across the field of view. Likewise, imaging the short axis of the IVC allowed for measurement of mediolateral displacement as well as the vessel's angle of collapse relative to vertical.

**Results:**

Seventy patients were enrolled over a 6-month period. The average diameter of the IVC was 13.8 mm (95% CI 8.41 to 19.2 mm), with a mean respiratory collapse of 34.8% (95% CI 19.5% to 50.2%). Movement of the vessel relative to the transducer occurred in both mediolateral and craniocaudal directions. Movement was greater in the craniocaudal direction at 21.7 mm compared to the mediolateral movement at 3.9 mm (*p* < 0.001). Angle of collapse assessed in the transverse plane averaged 115° (95% CI 112° to 118°).

**Conclusions:**

Movement of the IVC occurs in both mediolateral and craniocaudal directions during respirophasic ultrasound imaging. Further, collapse of the vessel occurs not at true vertical (90°) but 25° off this axis. Technical approach to IVC assessment needs to be tailored to account for these factors.

## Background

The increased presence of point-of-care ultrasound machines in the critical care setting has fueled attempts to use ultrasound as a noninvasive means of assessing volume status. Studies focusing on the sonographic measurement of the inferior vena cava (IVC) and its respirophasic change in diameter have demonstrated mixed results, but findings suggest that increase in respiratory variation of IVC correlates with low central venous pressure [1-4] as well as volume responsiveness in patients with septic shock [5,6]. On the opposite end of the spectrum, a decrease in IVC respiratory variation has been shown to correlate with volume overload in patients with renal failure and congestive heart failure [7,8].

**Figure 1:**
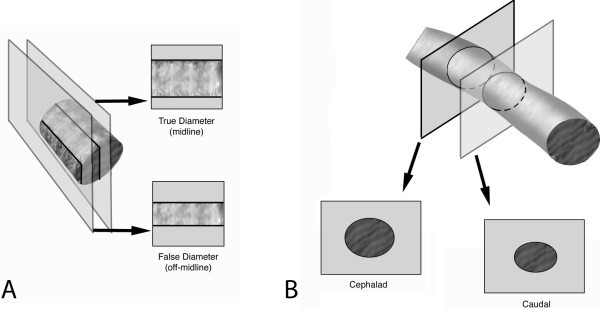
**Migration of inferior vena cava imaging planes.** Movement of the IVC during respiration introduces errors as the probe moves relative to the IVC from end inspiration to end expiration. (**A**) represents the imaging plane of a longitudinal view of the IVC. Probe movement off-midline results in measurement of a false diameter that is smaller than the true IVC diameter. As the IVC does not demonstrate uniform diameters along its course through the abdomen, error occurs in (**B**) transverse imaging where probe movement results in measurement of IVC diameter at different anatomic locations.

Despite a number of research studies focusing on sonographic evaluation of the IVC, there remains no standardization of the measurement technique [9] with researchers using both long-axis [5,6,10-15] and short-axis [7,15,16] imaging approaches. Both of these approaches have the potential to introduce error related to movement of the transducer during respiration and variations in normal anatomy. Transducer movement relative to the IVC secondary to respiratory mechanics as well as operator drift can result in comparing two different locations of the IVC during different times in the respiratory cycle. This type of error is affected by the imaging technique (long-axis or short-axis imaging). In the long axis (Figure 1A), movement off the midline of the IVC results in an artificial decrease in the measured diameter. In the short-axis (Figure 1B), movement in a craniocaudal direction could result in an artificial change in IVC diameter related to anatomic variation in size at different locations. Other aspects of IVC anatomy such as the axis of collapse [17] may also impact efforts to correlate respiratory variation and specific sonographic measurement techniques.

Standardizing the imaging methodology of IVC measurements could help improve the utility of this technique for determining fluid responsiveness. As a first step, we propose an analysis of the magnitude of movement of the IVC relative to the transducer during respirophasic imaging. The objective of this study was to quantify movements of the IVC relative to the axis of the transducer during quiet respiration with comparison of the movement in mediolateral and craniocaudal directions. We additionally sought to measure the vessel's axis of respiratory collapse during ultrasound imaging.

## Methods

Patients were prospectively enrolled as a convenience sample from a single urban academic emergency department with an annual volume of 85,000 patients. This study was approved by the local institutional review board with waiver of written informed consent.

Patients presenting to the emergency department with a variety of complaints were approached for inclusion in the study. Each ultrasound examination was performed by a single operator from a group of five emergency physician sonographers (senior resident, fellow, and attending level) all with experience in IVC ultrasound (>50 ultrasounds). All physicians involved in enrolling patients in this study underwent a minimum of 30-min didactic and 1-h structured hands-on dedicated to IVC imaging. Exclusion criteria included tachypnea, respiratory distress, and inability to fully visualize IVC from the right atrium to its bifurcation into right and left iliac veins. Sonographic images of the IVC were obtained using a standardized protocol consisting of B-mode images of the IVC in long axis and short axis obtained from an anterior transabdominal approach. Images were obtained using a 3.5-Mhz curved array transducer (Zonare Medical System, Mountain View, CA, USA) and recorded digitally. The long-axis view of the IVC consisted of images centered on the confluence of the hepatic veins and the IVC. Transverse views of the IVC consisted of views of the IVC immediately inferior to the hepatic veins. Recorded images included still images of both long-axis and short-axis as well as 10-seconds video clips recorded throughout a complete respiratory cycle of quiet respiration. The orientation of the ultrasound transducer relative to true horizontal was standardized using a leveling device attached to the ultrasound probe. The probe was held stationary 90° to horizontal throughout image acquisition.

**Figure 2:**
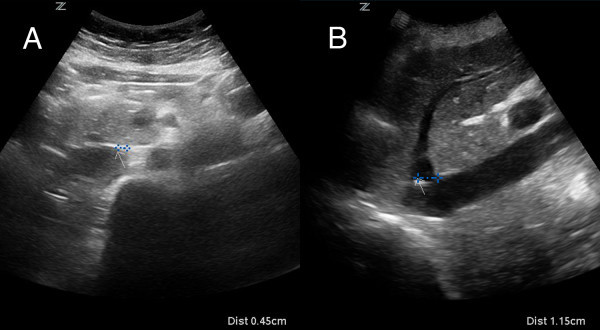
**Measurement of inferior vena cava movement.** An arrow is placed on the screen at the location of an identifiable landmark at end expiration in both (**A**) transverse and (**B**) longitudinal views. Movement during respiration is measured by placing calipers between the arrow and the location of the same landmark at end inspiration.

Maximum displacement of the IVC during the respiratory cycle was determined by identifying an anatomic landmark on the IVC and measuring the distance between its locations on the ultrasound screen from maximal expiration to maximal inspiration within a single respiratory cycle. This distance was measured with calipers at the time of the ultrasound exam by first placing an arrow on the screen at the lateral wall in transverse view (Figure 2A) or the insertion of hepatic veins in long-axis view (Figure 2B) at end expiration. The image was then frozen at end inspiration, and calipers were used to measure from the arrow, which remains stationary, to the new location of the same landmark. This still image displaying measured movement was recorded and stored digitally for later review. Additional measurements of the angle of collapse including both the major and minor axes were obtained using calipers and a linear angle ruler*.* The major axis is defined as the widest width of the IVC when maximally collapsed, as previously defined by Murphy and colleagues [17]. The minor axis is defined as the axis 90° to the major axis. The respiratory variation was calculated using the following equation:

(1) $$\frac{\text{Maximal diameter along minor axis}-\text{Minimal diameter in minor axis}}{\text{Maximal diameter along minor axis}}$$

Statistical analysis was performed using GraphPad Software, Inc (La Jolla, CA, USA) Data are presented as mean ±95% confidence interval unless otherwise specified. Comparison between measurements was performed using Student's *t* test.

## Results

Seventy patients were enrolled over a 1-year period from February 2010 to January 2011. Patients were 39% male with an average age of 40.2 (±21 years). Mean end-expiratory IVC diameter was measured along both major and minor axes. Diameter along the major axis averaged 27.4 mm (95% CI 19.9 to 34.9 mm), while the minor axis average diameter measured 13.8 mm (95% CI 8.41 to 19.2 mm).

**Figure 3:**
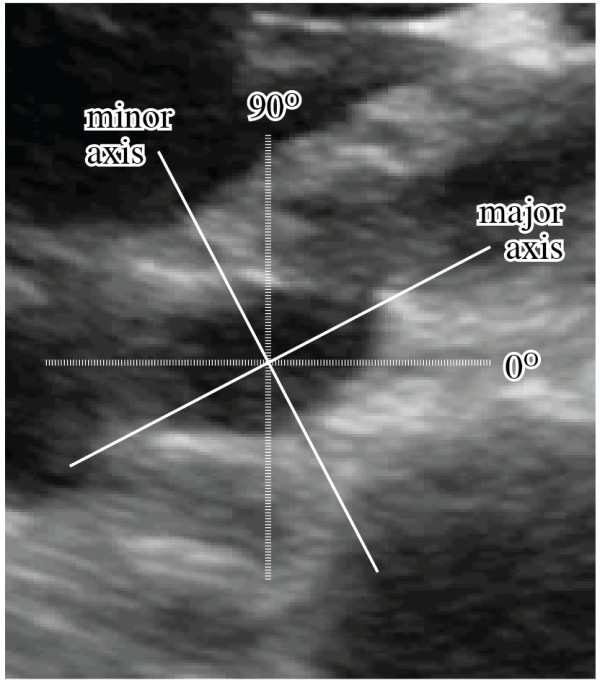
**Inferior vena cava axis of collapse.** The angle of collapse (minor axis) is measured relative to vertical (90°).

Major and minor axes were defined in the transverse plane by the observed angle of collapse relative to vertical (Figure 3). Defining true vertical as 90°, the primary angle of collapse of the vessel (minor axis) average was 115° (95% CI 112° to 118°). Collapse along this axis averaged 34.8% (95% CI 19.5% to 50.2%) during respiration.

**Figure 4:**
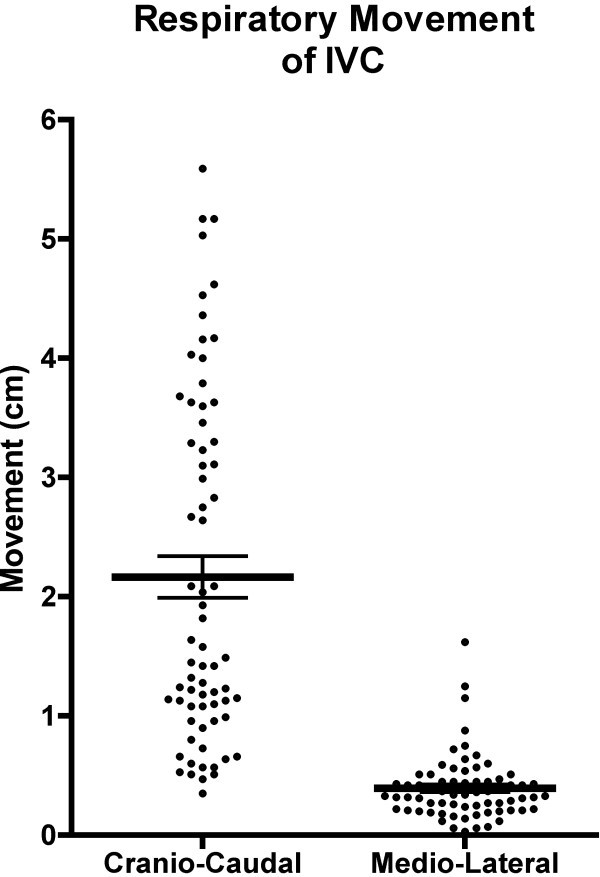
**Measured movement of the inferior vena cava during respiration.** Mean IVC displacements (with standard error of mean) are displayed with a scatter plot of data demonstrating a significantly greater movement in the craniocaudal direction (21.7 mm) than the mediolateral direction (3.9 mm).

Movement of the IVC was measured in both craniocaudal and mediolateral directions throughout the respiratory cycle (Figure 4). The mean caudal movement of the IVC during quiet inspiration averaged 21.7 mm (95% CI 18.3 to 25.1 mm). Sixteen percent (11 of 70) of patients demonstrated 40 mm or greater caudal movement during inspiration (range 3.5 to 55 mm). Lateral movement during respiration was significantly less with an average of 3.9 mm (95% CI 3.3 to 4.5 mm, *p* < 0.001). While the majority of patients showed lateral movement under 5 mm (54 of 70), three patients demonstrated movement of 10 mm or greater (range 0.3 to 16.2 mm).

## Discussion

Our data demonstrate that the IVC moves relative to the ultrasound transducer secondary to respiratory activity. Movement in either a craniocaudal or mediolateral direction has different implications depending on transducer orientation. Movement in a craniocaudal direction would be visible while scanning in the long axis of the vessel, but less obvious when imaging in the transverse plane. Conversely, mediolateral movement is evident while imaging in a transverse plane, but less so when imaging in long axis. In either plane, if the sonographer were not cognizant of this movement, measurement of the diameter at end inspiration and end expiration would occur at different anatomic locations, potentially introducing error in the measured respiratory variation.

It is not surprising that our results demonstrate a significantly greater movement of the IVC in a craniocaudal direction than a mediolateral direction, but the full clinical implications of these movements remain unclear as the magnitude of error would be related to anatomic changes in IVC course and diameter in each patient. It has been noted by some that the IVC is not a uniform diameter along its course through the abdominal cavity [9], but further research on anatomic variation of the IVC is required to determine if the larger craniocaudal movement corresponds to larger error. Even though our data demonstrate a drift of only 3.9 mm for a long-axis imaging technique, this represents a distance greater than half the radius of the vessel. In clinical use, this error could have a dramatic effect on the results of IVC volume assessment. If we assume a cylindrical vessel of 27 mm in diameter, a 3.9-mm drift off midline would result in a measured new diameter of 12.9 mm, erroneously indicating a greater than 50% respiratory variation in diameter. This example likely overestimates the variability by assuming a cylindrical vessel but highlights the need for further research into what the actual change in diameter is with drift off midline. Additionally, while it is clear that in the long axis the drift will reliably cause a measured decrease in diameter, the effect of short axis views that drift superior or inferior is unknown without knowing the change in diameter along the vessel's course.

The reliability of IVC measurements are further complicated by the angle of collapse that is not truly vertical. Murphy et al. described the effects of IVC diameter with changes in volume status as measured by sequential computed tomography scans, finding that the vessel distends primarily along a defined minor axis that averaged 116° from midline [17]. Our study reveals similar findings with a minor axis of 115° along which the vessel demonstrated its greatest respiratory variation in diameter. This angle of collapse is apparent on short-axis imaging of the IVC and can be compensated for during IVC measurement. The angle of collapse is not visible when the IVC is imaged in long axis, making comparisons between long axis and short axis more difficult.

The inherent error related to respirophasic IVC measurements we describe may help explain findings in the recently published literature. Fields et al. recently studied interrater reliability of IVC measurements by bedside clinician sonographers [11]. While they found strong agreement between sonographers for measured maximum diameter of the IVC, the agreement was markedly decreased for measurement of IVC collapse. This study utilized M-mode tracings for measurement of collapse and was not designed to evaluate the difference between long-axis and short-axis imaging approaches. The determination of which technique has the least inherent error is difficult without further research that examines the magnitude of change in diameter of the IVC along its course.

### Limitations

Our measurements were taken in patients without acute distress during quiet respiration. It is possible that IVC movement in these cases does not reflect the movement that might be expected in patients with respiratory difficulty or other compromise. The generalizability of our findings to other sonographers is limited by the relatively few numbers of sonographers that obtained images for this study. Determining the movement of the IVC during ultrasound imaging is effected by individual sonographer technique as movement of the IVC is multifactorial, influenced not only by patient factors (caudal displacement of the IVC with movement of the diaphragm), but also by sonographer factors (compensatory movements by the sonographer to compensate for the abdominal or chest wall movement).

We have limited information on the clinical status of patients in this study (diagnosis, volume status, etc.), only a review of the technical aspects of their imaging studies. It therefore remains a possibility that our patient population does not reflect the general population as a whole.

## Conclusion

During ultrasound imaging of the IVC, there is significant movement of the vessel relative to the scanning probe, with the greatest movement occurring in a craniocaudal direction. The axis of greatest collapse of the IVC occurs not along the anteroposterior diameter of the vessel, but at an angle of 115°. Sonographic assessment of respiratory variation in IVC diameter should be tailored to account for these factors.

## Competing interests

The authors declare that they have no competing interests.

## Authors’ contributions

DB conceived and designed the study, coordinated the study activities, and drafted the manuscript. RG participated in the study design, statistical analysis, and manuscript revision. DR participated in the study design as well as subject enrollment. BC and MD participated in the subject enrollment. All authors read and approved the final manuscript.
